# Successful transplantation of kidneys from deceased donors with terminal acute kidney injury

**DOI:** 10.1080/0886022X.2019.1590209

**Published:** 2019-03-26

**Authors:** Piotr Domagala, Lukasz Gorski, Michal Wszola, Rafal Kieszek, Piotr Diuwe, Piotr Goralski, Jakub Drozdowski, Agata Ostaszewska, Jolanta Gozdowska, Michal Ciszek, Janusz Trzebicki, Magdalena Durlik, Leszek Paczek, Andrzej Chmura, Artur Kwiatkowski

**Affiliations:** a Department of General and Transplantation Surgery, The Medical University of Warsaw, Warsaw, Poland;; b Department of Transplant Medicine, Nephrology and Internal Medicine, The Medical University of Warsaw, Warsaw, Poland;; c Department of Immunology, Transplantology and Internal Medicine, The Medical University of Warsaw, Warsaw, Poland;; d Department of Anaesthesiology and Intensive Care, The Medical University of Warsaw, Warsaw, Poland

**Keywords:** Acute kidney injury, deceased donors, expanded criteria donors, kidney graft survival, kidney transplantation

## Abstract

**Background:** There are many doubts with regards to accepting deceased kidneys with acute kidney injury (AKI) for transplantation.

**Purpose:** The aim of this study was to present the 5-years outcome of kidney transplantation cases where deceased donors developed AKI before organ procurement.

**Methods:** Two hundred twenty-six deceased renal transplants were analyzed. Data regarding donors and recipients were collected. Terminal AKI was defined as terminal serum creatinine concentration higher than 1.99 mg/dL and 66 such cases were diagnosed. All kidney transplant recipients were followed for 60 months.

**Results:** AKI group presented more episodes of delayed graft function (DGF) compared to the non-AKI group (56% vs 35%, *p* < .05). No differences were observed between the groups in the rate of acute rejection episodes, kidney function as well as patient and graft survival.

**Conclusions:** Transplants with AKI present more often DGF and comparable graft survival to transplants without AKI. Kidneys with AKI can be a valuable source of organs provided attentive selection and appropriate care of deceased donors.

## Introduction

Kidney transplantation is the first choice treatment for end-stage renal disease patients. The main obstacle in offering this treatment to everyone who needs it is an organ deficiency. There is still a discrepancy between the number of patients on the waiting list for kidney transplantation and the number of performed transplants [1]. The needs exceed the capabilities of supply. Strategies for increasing transplant availability include using expanded criteria donor (ECD) organs, donor after cardiac death organs, dual kidney transplantation, living organ donation, and experiments with artificial organs.

With the beginning of this century on the basis of Scientific Registry of Transplant Recipients a definition of ECD was obtained. An ECD definition is based on the following criteria: a donor older than 60 years or a donor older than 50 years with at least two of the subsequent: hypertension, stroke as a cause of death, or serum creatinine greater than 1.5 mg/dL [2]. Recipients of kidneys procured from ECDs have a risk of graft loss that is 1.7x higher than that of recipients of kidneys obtained from ‘ideal donors’ (10–39 years old, without hypertension, stroke not the cause of death, and creatinine concentration lower than 1.5 mg/dL) [3,4]. As this simple tool seemed to not be sufficient for deceased donor kidney evaluation, a novel tool called the kidney donor risk index (KDRI) has been implemented during the last decade [5]. Although the KDRI can be complex and difficult in everyday use, it has been proven to be more relevant in kidney assessment [6]. Despite these improvements in donor kidney assessment, many patients die on the kidney waiting list each year due to insufficiency in organ supply [7]. Thus the transplant community could benefit from the utilization of kidneys from high risk donors.

The evaluation of expanded criteria kidneys should take into consideration the balance of risk between the transplantation of a kidney that is not considered ideal and waiting for a better kidney, resulting in a higher probability of death [8]. The aim of accurate kidney evaluation is to increase the number of transplantable organs from the donor pool. Recent improvements in kidney assessment are probably the main reason for the doubling of the kidney transplant discharge rate (from 0.1 to 0.2) that occurred during the first decade of this century [7]. The more potential deceased kidney donors are considered to become a real donor, the higher rate of kidneys discharge is observed. Kidneys are disqualified from transplantation after the procurement, during the storage process, based on perfusion parameters, macroscopic inspection or other factors.

A high terminal creatinine concentration was historically a relative contraindication to kidney donation and transplantation because of the expected poor outcome. Nowadays, in the era of organ shortage, donors with a terminal creatinine concentration higher than 3.5 mg/dL, donors presenting with oligoanuria and even donors on hemodialysis become a matter of interest for the transplant community. After brain death, most donors are exposed to hypovolemia, hypotensive shock, hypoxic-ischemic injury, cytokine release, nephrotoxic agents, disseminated intravascular coagulation, and infections [9]. These factors can lead to renal damage and acute kidney injury (AKI) in donors without a preexisting history of kidney disease. Kidney injury may be completely reversible after kidney transplantation when the underlying cause of this injury is treated [10].

There is confusion in the nomenclature and classification of AKI. Many authors use a different definition of AKI to suit their purpose. The RIFLE criteria consists of 5 stages of renal injury (‘risk, injury, failure, loss, and end-stage renal disease’) and was proposed by the Acute Dialysis Quality Initiative group [11]. This classification was intended to standardize the severity of AKI. A modification of the RIFLE criteria was introduced by the Acute Kidney Injury Network (AKIN) [12]. The AKIN group proposed a standardized definition of AKI and established 3 stages of AKI for native kidneys but not for donors following brain death. In the past decade, few authors have adapted the AKIN criteria for donor kidney evaluation and prediction of renal allograft outcome. Rodrigo et al. [13] were first to adopt the RIFLE criteria to evaluate AKI in donor kidneys following brain death. They concluded that the RIFLE criteria are useful for proper AKI diagnosis and for epidemiological stratification of kidney donors with renal dysfunction [13].

This study examined organ donors presented AKI as a potentially valuable origin of kidneys for transplantation. The aim of this study is to present, that renal transplants from donors with AKI may have adequate renal function 5 years after transplantation.

## Materials and methods

A retrospective single center cohort study was conducted including all consecutive kidney transplants from deceased donor after brain death (*n* = 226).

Donors’ and recipients’ data as well as the evidence about the methods of kidneys’ storage were collected.

### Donors

There were 128 deceased donors diagnosed with brain death, aged from 12 to 77 years (mean = 46 years) that had been hospitalized in an intensive care unit for 1 to 14 days (mean stay = 4.5 days). The mean donor terminal serum creatinine concentration was 1.83 mg/dL (range, 0.40–6.04 mg/dL). Vasopressors, most commonly dopamine, were administered in 84% of donors. More than 30% of the donors fulfilled the expanded criteria (Table 1). The terminal creatinine concentration more than 1.99 mg/dL in a donor without a previous history of chronic kidney disease was defined as the terminal AKI. All of the donors had normal urine output. Almost all kidney donors, including those classified as AKI donors, had normal serum creatinine concentrations (<1.5 mg/dL) before the development of kidney injury.

**Table 1.** Donor characteristics (*n* = 128).

Age (y)	46
Male / Female (%)	63 / 37
Hypertension (%)	29
Serum creatinine concentration (mg/dL)	1.83
Expanded criteria donors (%)	32
Body mass index (kg/m^2^)	25.7
Hypotension (%)	63
Cardiac arrest (%)	26
Intensive care unit stay (days)	4.5
Donor vasopressors administration (%)	84

### Kidney recipients

A total of 226 patients with end stage renal disease received a renal transplant from a deceased donor in the period between 2010 and 2011. The recipients age ranged from 19 to 76 years (mean = 47 years), they were predominantly male (62% males), and their mean body mass index (BMI) was 24 kg/m^2^. The mean duration of dialysis therapy prior to transplantation (hemodialysis in 93%) was over 44 months. Most of the recipients suffered from hypertension (85%) and 14% suffered from chronic coronary disease.

### Kidney procurement, preservation, and transplantation

Immediately prior to surgery, donors were given heparin (25 000 IU) and a broad-spectrum antibiotic as a prophylactic. Procurement of kidneys was typical and *in situ* perfusion was performed using University of Wisconsin solution. Immediately after the organ recovery procedure and cooling to 4 °C, 21 kidneys (9%) were placed in a thermally stable container filled with preservation solution and 205 kidneys (91%) were allocated to a system for hypothermic machine perfusion (LifePort, Organ Recovery System, IL, USA). The decision of which type of storage was used was made depending upon cassette and perfusion fluid availability. Whenever possible, AKI donor kidneys were stored using machine perfusion. All 226 kidneys were transplanted to recipients from the National Waiting List. The surgical procedure was typical and a mean cold ischemia time was over 29 h.

### Recipient observation

The patient and graft survival, as well as the delayed graft function (DGF) were analyzed. Recipient kidney function was estimated with serum creatinine concentration and estimated glomerular filtration rate (eGFR) at 12, 24, 36, 48, and 60 months after transplantation. DGF was determined when there was the necessity for dialysis treatment during the first week after transplantation. eGFR was calculated with the use of the Modification of Diet in Renal Disease formula (MDRD).

Most patients received triple drug immunosuppressive therapy that included steroids, cyclosporine or tacrolimus, and mycophenolate. In cases of strong immune responders, induction immunosuppressive treatment was added (basiliximab).

### Statistical analysis

Graft survival was established as an endpoint. Secondary endpoints included: DGF, acute rejection and kidney graft function. Patient death reflects the graft loss.

Differences between groups were assessed by means of Chi-square or Cochran–Mantel–Haenszel tests. The Student’s *t*-test and Wilcoxon test were applied for differences between means and medians, respectively. The critical level for hypothesis testing was set at *p* = .05. Statistical analysis was performed using SAS software (Version 8.2; SAS Institute Inc., Cary, NY, USA).

## Results

During the period included in this study, 226 patients were transplanted in our department. For the purpose of this study, two groups were selected: recipients of kidneys from donors with terminal creatinine concentrations lower than 2.0 mg/dL (non-AKI group; *n* = 160) and recipients of kidneys from donors with terminal creatinine concentrations higher than 1.99 mg/dL (AKI group; *n* = 66).

Unexpectedly, the donors in the AKI group were younger (42 years vs 47 years; *p* = .01). The percentage of male donors in the AKI group was higher (80% vs 55%; *p* = .0001). The donors from the AKI group had a significantly higher BMI in comparison to the non-AKI group (26.9 ± 3.9 kg/m^2^ vs 25.3 ± 5.2 kg/m^2^; *p* = .015). Mean serum creatinine concentration was 1.03 ± 0.42 mg/dL in non-AKI donors and 3.82 ± 1.15 mg/dL in the AKI group at the time of kidney procurement (Table 2). The causes of donor death included cerebrovascular accident (57.5%), central nervous system trauma (34.9%) and others (7.5%). There were no differences between groups.

**Table 2.** Characteristics of acute kidney injury-(AKI) and non-AKI groups.

	non-AKI (*n* = 160)	AKI (*n* = 66)	*p*
Donor age (y ± SD)	47 ± 15	42 ± 15	.01
Donor sex (male/female) (%)	55/45	80/20	.0001
Donor hypertension (%)	26.6	18.7	ns
Donor BMI (kg/m^2^ ± SD)	25.3 ± 5.2	26.9 ± 3.9	.015
Donor hypotension (%)	63	63	ns
Donor cardiac arrest (%)	24	30	ns
Donor intensive care unit stay (days)	4.5	4.8	ns
Donor vasopressors administration (%)	83	89	ns
Donor serum creatinine (mg/dL ± SD)^a^	1.03 ± 0.42	3.82 ± 1.15	<.00001
Expanded criteria donor (%)	32	33	ns
Donor urine output (mL/h ± SD)^a^	173 ± 104	154 ± 97	ns
Hypothermic machine perfusion (%)	89	95	ns
Cold ischemia time (h ± SD)	29.1 ± 8	30.5 ± 8	ns
Mean human leukocyte antigen mismatch	3.6	3.4	ns
Recipient age (y ± SD)	48 ± 15	47 ± 14	ns
Recipient sex (male/female) (%)	64/36	56/44	ns
Recipient BMI (kg/m^2^ ± SD)	24.5 ± 3.8	24.2 ± 3.2	ns
Recipient hemodialysis treatment (%)^b^	94	90	ns
Recipient hypertension (%)	84	87	ns
Recipient chronic coronary disease (%)	11	20	ns

a At organ procurement.

b Prior to transplantation.

BMI: body mass index; ns: not significant

There was no difference between non-AKI and AKI kidneys regards the method of storage (89% of non-AKI and 95% of AKI kidneys were stored in hypothermic machine perfusion; *p*=NS). The mean flow in non-AKI kidneys group was significantly higher in comparison to AKI kidneys group the first hour of perfusion (112 mL/min vs. 97 mL/min; *p* = .026). The non-AKI kidneys group has also a tendency to have lower resistance compared to the AKI kidneys group the first hour of perfusion (0.26 mmHg × min/ml and 0.35 mmHg × min/ml; *p* = .057). There were no differences in the flow and the resistance between non-AKI and AKI kidneys regards next measurements during perfusion.

There were no differences in panel reactive antibody (PRA) and in human leukocyte antigen (HLA) mismatch between groups (Table 2). No differences in the requirement for immunosuppressive therapy were observed between the AKI and non-AKI groups. The reasons of the end stage renal disease (ESRD) included: glomerulonephritis (29.7%), congenital disease (22.5%) diabetes mellitus nephropathy (19.3%), hypertension nephropathy (7.5%) and others. There were no differences in the cause of ESRD between groups.

**Figure 1.**
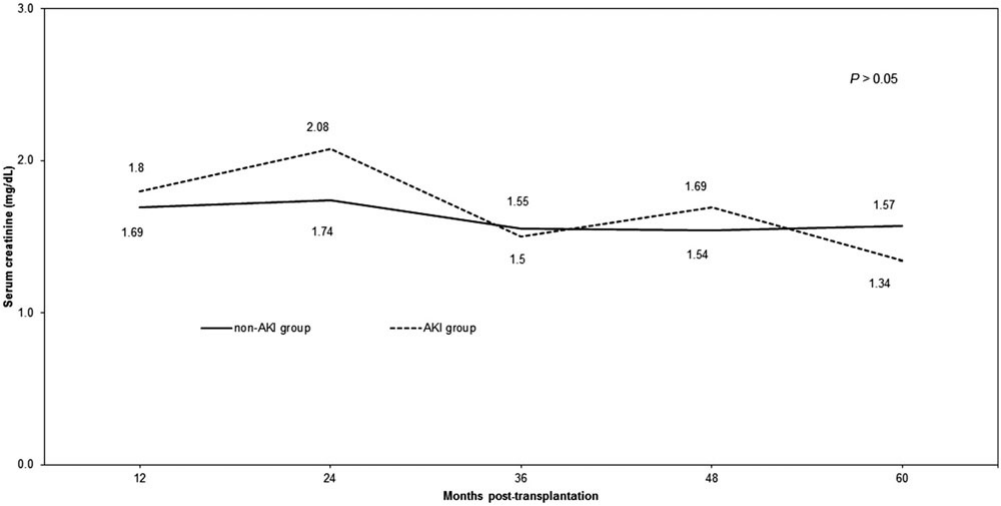
Mean serum creatinine in the acute kidney injury (AKI) group and the non-AKI group.

**Figure 2.**
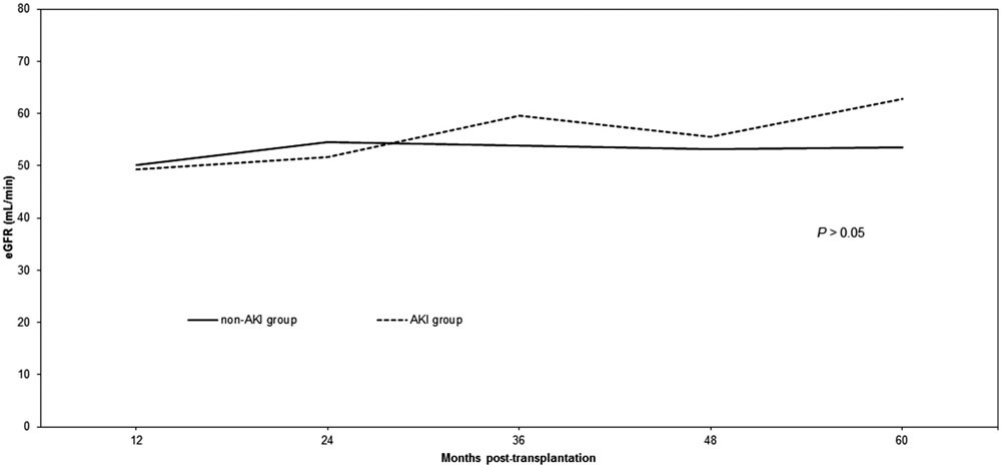
Mean estimated glomerular filtration rate (eGFR), according to the modification of diet in renal disease formula, in the acute kidney injury (AKI) group and the non-AKI group.

More episodes of DGF were observed in the AKI group (56% vs 35%; *p* = .004). There were no differences in the rate of biopsy-proven acute rejection episodes in the first year post-transplantation (Table 3). Graft survival did not differ between the groups significantly during the follow-up period. The average serum creatinine concentration did not differ between the AKI and non-AKI groups at 12, 24, 36, 48, or 60 months post-transplantation (Figure 1). Accordingly, the average eGFR did not differ between the AKI and non-AKI groups (Figure 2).

**Table 3.** Results of kidney transplantation in acute kidney injury (AKI) and non-AKI groups

	non-AKI (*n* = 160)	AKI (*n* = 66)	*p*
Delayed graft function (%)	35	56	.004
Hospital stay (days)	17.7	19.6	ns
Biopsy proven acute rejection episodes to 1 year post-transplantation (%)	12.6	10.9	ns
Graft survival (%):			
12 months	94	90	ns
24 months	92	89	ns
36 months	89	88	ns
48 months	87	85	ns
60 months	84	81	ns

## Discussion

In this current era of a growing number of patients awaiting a kidney, identifying an additional pool of donor kidneys that is suitable for transplantation is urgently needed. In this single center analysis, we found that the transplantation of kidneys procured from donors with AKI performed similarly to organs obtained from donors with normal kidney function. According to our findings, donors from the AKI group were predominantly male, were younger, and had a higher BMI. The issue of age could be explained that older deceased ICU patients with acute kidney injury are probably not taken into consideration as potential organ donors and are not referred to transplant coordinator. Higher BMI may be explained by the fact that obese people have a bigger chance of developing acute renal failure (ARF). There were no significant differences in the occurrence of biopsy-proven acute rejection episodes. The rate of DGF was significantly higher in the AKI group compared with the non-AKI group (56% vs 35%; *p* = .004). The concentration of serum creatinine was 1.69 versus 1.80 mg/dL at 12 months post-transplantation, and 1.57 versus 1.34 mg/dL at 60 months post-transplantation, in the non-AKI and AKI groups, respectively. These differences in serum creatinine were not statistically significant. Graft survival did not differ significantly between the two groups.

Despite few published research dealing with transplantation of kidneys with AKI, there is apprehension towards the procurement of kidneys from donors with AKI [14]. Most previous studies are retrospective single center studies and some are registry studies, but there remains a lack of evidence from randomized controlled studies. There are no generally applicable guidelines regarding evaluation, storage methods, diagnostic testing, or allocation policies for such kidneys. Different definitions of AKI have been identified and tested, making comparisons between the results of different studies difficult.

Kidneys procured from AKI donors have been previously reported to be more prone to develop DGF [15,16] and this was also observed in the current study. There is no evidence that AKI in donor kidneys has an impact on acute rejection episodes in recipients [17]. Many studies have presented similar survival rates for kidneys from AKI and non-AKI groups, despite using different definitions of AKI [18]. Zuckerman et al. [19] reported no differences in 12-month kidney survival (92%) in AKI and non-AKI groups using a similar definition of AKI as was used in the current analysis. Other analyses from the same center have shown that 60-month graft survival in AKI and non-AKI groups was 71% and 78% (*p*=ns), respectively [16]. There was also no difference in kidney function between groups. In addition, Kumar et al [20] showed that 3-year graft survival was 90% in the AKI group (named as ARF), and this rate of survival was similar to that observed among standard criteria donor (SCD) kidneys and was higher in comparison to ECD kidneys. Similarly, data presented by colleagues from Brazil has demonstrated similar 5-year graft survival in AKI and non-AKI groups of 81% and 84% (*p*=ns), respectively [21].

On the other hand, some authors have reported differences in survival of kidneys procured from donors with AKI. Kolonko et al. [22] reported that long-term kidney graft survival was significantly lower amongst kidneys procured from donors with AKI. These findings are similar to those of Boffa et al. [23], who analyzed more than 10 000 transplanted kidneys from the United Kingdom Transplant Registry, of which 17% met the criteria of AKI. The 5-year survival of kidneys from the non-AKI and AKI groups was 76% and 78% (*p* = .009), respectively. They also reported that kidney function was slightly worse in recipients of AKI kidneys; however, the eGFR was only measured during the first year after transplantation. Nevertheless, these authors suggested that donors with AKI are a viable source of organs and that these kidneys should not be discarded. Jacobi et al. [24] underlined that kidneys retrieved from AKI donors should not be discarded, even though 1-year allograft survival rates were lower in recipients of an ECD kidney with AKI. Similarly, Deroure et al. [25] pointed out that kidneys from ECD donors with ARF should not be immediately discarded and should be considered for transplantation with caution. Different findings were presented by Klein et al. [26], who reported no difference in 1-year kidney graft survival irrespective of whether the kidney was procured from an SCD or ECD with or without ARF.

In the current study, we used a simple cutoff point to determine the presence of AKI, and we did not analyze this group according to severity of AKI. Several researchers have attempted to evaluate whether the severity of AKI has an influence on the rate of kidney transplantation success. However, during long-term graft survival follow-ups, it is often impossible to proceed with such an analysis due to the small number of cases [23,27]. Lee et al. [27] demonstrated that there was no difference in the development of DGF, acute rejection episodes, or kidney function between kidneys at different stages of AKI (as defined by the AKIN criteria). Ali et al [28] also reported that AKIN category did not influence kidney graft survival, although the number of cases in this long-term observation was small.

It is obvious that kidney injury within the donor body is not the only important factor for success in organ transplantation. Cold ischemia time (CIT) has been widely explored as a factor that influences short-term kidney allograft function. Xia et al. [29] analyzed the impact of prolonged CIT in combination with AKI on short and long-term outcome of kidney transplantation. Their data showed that prolonged CIT does not negatively affect graft function and survival when the kidney was procured from a donor with AKI. Exploring the field of exploitation of AKI grafts, the data reported by Heilman et al. [30] seem to bring a breath of novelty into the discussion. This group analyzed pathology, as well as gene expression profiles, in addition to typical clinical outcomes. They did not find remarkable differences in acute or chronic histologic changes according to the Banff classification between the AKI and non-AKI groups. The researchers found significant up-regulation of several genes, including 70% of kinase molecules, 75% of transcriptional regulator molecules, and 70% of genes associated with cell death, in the AKI group compared to non-AKI group in kidney biopsies sampled during the first month post-transplantation. In contrast, no differences were found when the same analysis was performed at 4 months post-transplantation. It is believed that all genetic markers of AKI-induced injury that presented during the first month had resolved by the fourth month.

In our opinion donors with AKI should be considered as potential kidney donors when initial (at the time of admission) kidney function was normal, the donor did not have any medical history of kidney disease and the perfusion parameters (flow and resistance) of the kidney improved during machine perfusion.

Our analysis confirms that kidneys with AKI are prone to develop DGF and have comparable long-term results with non-AKI grafts. It is remarkable in our analysis, that almost 91% of all grafts were stored with the use of machine perfusion (89% in non-AKI and 95% in AKI group). In referenced studies there was no information about the type of storage, in some centers only cold storage preservation was used and in others the machine perfusion was used only for grafts with acute kidney injury. In contrast to other studies we presented results of machine perfusion during organ storage – the flow and the resistance (see above). In our opinion the machine perfusion should be used in every case of procured graft with acute kidney injury in order to assess the organ and presumably to improve results of transplantation.

Our findings have limitations. This was a single center retrospective analysis. Many factors affect kidney graft function and survival, including donor and recipient details, methods of organ preservation, and post-transplantation treatment, making it very difficult to isolate any individual factor that influences the success of kidney transplantation. The terminal serum creatinine concentration is not an ideal kidney function marker and can be affected by many factors. The definition of AKI used in this study was simplified and did not divide the AKI group according to severity of AKI. In our opinion, due to the small number of available kidneys with AKI (*n* = 66), it was better to compare all AKI kidneys to all non-AKI kidneys. Nevertheless, our analysis included a 5-year follow-up what increases the relevance of the conclusions.

In summary, we arrived at the conclusion that selected deceased donors with AKI are a significant origin of kidneys that should be used by the transplant community. Many kidneys are discarded because of an elevated terminal serum creatinine concentration. Although AKI kidneys are more likely to have DGF, they have comparable survival and function in comparison to non-AKI kidneys. Donor characteristics, machine perfusion parameters, and kidney biopsy findings are important tools to support the decision to offer kidneys with AKI. Kidneys with AKI can expand the organ pool and outcomes can be optimized provided attentive selection and appropriate care of deceased donors.
